# Integrated Family Approach in Mental Health Care by Professionals From Adult and Child Mental Health Services: A Qualitative Study

**DOI:** 10.3389/fpsyt.2022.781556

**Published:** 2022-04-28

**Authors:** Hanna Stolper, Karin van Doesum, Majone Steketee

**Affiliations:** ^1^Department of Psychology Education and Child Studies, Erasmus University Rotterdam, Rotterdam, Netherlands; ^2^Department of Clinical Psychology, Radboud University Nijmegen, Nijmegen, Netherlands

**Keywords:** integrated family approach, adult and child mental health services, parental mental disorder, infants and early childhood, transmission of psychopathology, family focused practice, qualitative study

## Abstract

**Objective:**

A multiple case-study in which each case was evaluated by adult and child mental health professionals who used an integrated family approach in their treatments. In this approach, treatment focuses on the mental disorders of the parents as well as on the development of the young child and family relationships. This study evaluated the experiences of professionals from adult and child mental health services using this approach. The aim of the study is identifying key elements of this approach, processes involved in treatment, and barriers to its success, with the aim of contributing to the development of practice based integrated mental health care for the whole family.

**Background:**

Parental mental disorders have an impact on parenting and child development. To stop detrimental cascade effects and prevent parent and child from being caught up in the intergenerational transmission of psychopathology, an integrated family approach in mental health care is needed. Methods: A qualitative case study design using a grounded theory approach. Data were collected through 19 group interviews of professionals (*N* = 37) from adult and infant mental health teams who worked together in the treatment of a family.

**Results:**

Professionals from the two services were comfortable coping with complexity and felt supported to perform their treatments by staying in touch with each other in multi-disciplinary consultations. They indicated that by attuning the treatment components to each other and tailoring them to the capabilities of the family, their treatments had more impact. A flexible attitude of all involved professionals and commitment to the interest of all family members was essential.

**Conclusion:**

According to professionals, treatment with an integrated family approach in mental health care is of value for families by addressing the distinct roles, positions and relationships, by implementing a flexible complementary treatment plan, and by empowering professionals by multi-disciplinary consultations.

## Introduction

In the Netherlands, an integrated family approach in mental health care has been developed that involves the parent with a mental disorder as well as the young child and their relationships within the family in order to prevent the intergenerational transmission of psychopathology from the parent to the child. Epidemiological research provides evidence that children of parents with a mental disorder are at serious risk of developing a mental disorder of their own during their lifetime (1, 2). The prevalence of mental disorders in such children ranges from 41 to 77% for the whole diagnostic spectrum (3). The association between mental disorders in parents and their development among children is complex and depends on a number of interacting risk and protective factors (3–5). The risks are more prominent during pregnancy and early life due to the vulnerability of the fetus and the great dependence of the infant on the environment. In this crucial phase of brain development, the brain is vulnerable to stress, and the exposure to stress early in life is associated with later psychopathology (6, 7). The establishment of a secure attachment relationship with caregivers is another critical development during the first year of life, which provides an important foundation for subsequent cognitive and social emotional development [for instance, see (8)]. In addition, a secure attachment style is mentioned as a protective factor against the development of psychopathology (9–11). Therefore, mental health care that integrates treatment of parents with a mental disorder and their young children is needed (5).

Parenting is likely to be more challenging for parents with a mental disorder because there is a risk of reactivating or exacerbating the symptoms (12–14). Although parenthood can be a motivator for parents with mental disorders to manage their symptoms more effectively (15), there is a great deal of evidence that suggests parenting can be affected by a mental disorder (13, 16, 17). For instance, depression is associated with such parental behaviors as unresponsiveness, intrusiveness, hostility, and a high level of expressed negative emotions (18).

According to Sameroff's (19) transactional model, the bidirectional nature of the parent-infant relationship over time affects both parent and child. For instance, if the parent is unpredictable in emotional availability and stimulation, the young child is at risk of developmental delays, insecure attachment styles, and challenging behavior. This in turn will make parenting more stressful and less satisfying with the additional risk of aggravating the parents' psychiatric symptoms. Therefore, in addition to the treatment of parental mental disorders, mental health services should include parenting and the evolving parent-child relationship to prevent and repair negative parent-infant interaction patterns (20, 21).

To prevent parents and children from negative cascade effects and being caught up in the cycle of intergenerational transmission of mental disorders and adverse outcomes, a paradigm shift from an individual model to a family centered model in psychiatry is needed (13, 22). However, treatment with a focus on the whole family is not common in adult mental health services for various reasons (13, 23). Worldwide, mental health services for adults and children are mostly separated, and professionals are educated in one of these two areas of clinical practice. An individual perspective on mental disorders and symptoms in adult mental health care results in a limited view that excludes the role of patients as parents, their dependent children, and the developing parent-child relationship (24, 25). In addition, child mental health services focus one-sidedly on the problems of the child and do not incorporate the mental health problems of the parents in treatment (26, 27). The split between adult and child mental health services makes it more difficult to meet the needs of parents, their young children, and the evolving relationship at the same time. Collaboration between adult and child mental health services is recommended by several researchers (22, 23, 28, 29).

An integrated family approach in the practice of mental health care involves multi-disciplinary treatment for the family as a whole. Treatment using this approach offers parents with a diversity of mental disorders and their young children a combined treatment addressing current problems in different domains within the family, namely the mental disorder(s) of the parent(s), the partner relationship, parenthood and family life, the parent-child relationship, and the child's mental or relational disorder. The aim of this collaborative integrated family approach is to increase the quality and efficiency of the treatment for parents and their young children, to improve their relationships, and to ameliorate the risk of intergenerational transmission of psychopathology or other adverse outcomes.

This combined treatment is conducted by professionals from adult and child mental health services, who collaborate closely by tailoring treatment components to the capabilities of the family, and their social and socio-economic context. These professionals carried out their treatments according to their own expertise, parallel or integrated, but always in tune with the other treatments. Therefore, they meet up regularly in a multidisciplinary consultation.

A prominent feature of families considered for integrated treatment is a complexity of interacting problems which often result in negative cascading effects. Therefore, treatment using an integrated family approach is not conducted according to a standard fixed program, but is tailored to the specific needs of the individual family. For instance, families in which there are conflicts between the parents receive couples therapy, while in other families there is a need for home treatment because of unpredictable, or lack of, daily routines. Professionals jointly determine in their multidisciplinary consultations which targets of intervention should be prioritized to initiate positive cascade effects, in which sequence, in which timeframe, and by which professionals. Expertise from adult and children's mental health services is brought together for the benefit of the whole family.

The integrated family approach is closely related to family-focused practice (FFP), which is an umbrella term for preventive and supportive interventions within adult mental health care services in which attention is paid to the family members of the patient, especially the children (22). An example of this approach is psycho-education regarding a parent's mental disorder for both the partner and the children. The difference between FFP and the integrated family approach is that the latter incorporates the parent with the disorder, the young child, and their relationships within the family in their treatment, and therefore integrates treatments by adult and child mental health services professionals. However, the aims of treatment using the integrated family approach and FFP are identical, namely the prevention of intergenerational transmission of psychopathology.

There are no studies whereby such integrated treatment and collaboration between professionals from adult and child mental health services has been evaluated. Thus, the main aim of this study is to gain insight into this approach by identifying which key elements of this approach and which processes that occur during treatment contribute to treatment success and what barriers exist to treatment success. In addition, we hope to inspire and motivate professionals and mental health services to treat the family in an integrated way so that families as a whole benefit from treatment. This study is part of a broader study that also examines the experiences of the parents and the effects of this treatment; however, the data collection for this has not yet been completed. The research questions posed in this study were: First, what do professionals indicate as key elements of success in an integrated family approach, and which processes amongst professionals emerged, in treatment of parents and their young children? Second, what do professionals identify as the benefits for the whole family? Third, what challenges or barriers did professionals experience that posed a threat to the success of treatment?

## Methods

### Design

In this multiple case-study of 19 families, we evaluated with professionals of adult mental health service (AMHS) and child and adolescent mental health service (CAMHS) their experiences with the use of an integrated family approach in these 19 cases. We adopted a qualitative design using a grounded theory approach. This approach is suitable when no previous research has been done about the object of study (30). As far as we know there is a lack of knowledge of experiences of professionals using a multi-disciplinary family approach involving both adult and child mental health services. The grounded theory approach is an inductive method to derive theory through systematic collection and analysis of data (31). In line with the interpretive grounded theory approach, researchers were engaged with and played an active role in interpreting the data, resulting in a theory about key elements, and processes involved in an integrated family approach grounded in the data (32), as well as barriers to this success.

We conducted group interviews, with the professionals (*N* = 37) who were involved in these 19 cases. Group interviews have the potential to create a process among professionals wherein they can share and compare their views and experiences in a conversation (33). This can lead to a reflective stance of the professionals and increases the chance of more differentiated and deepening information about experiences with integrated family treatment and reflecting on factors contributing to and obstructing successful treatment compared to individual interviews.

### Data Sampling

The data were collected through group interviews with professionals who were actually involved in the treatment of the 19 selected families. Each group interview focused on one of the 19 cases. In the group interview, the treatment the family received was evaluated by all professionals who played a substantial role in that treatment. Ethics approval was granted by the Medical Ethics Review Board at the University Medical Centre of Utrecht in the Netherlands (18-186/C). Parents of the families were asked by their therapist if they would be willing to participate in the study. The selection of the 19 family cases was based on the following criteria: adult patients with a mental disorder according to the DSM-5 and a young child up to 6 years, with relational problems or other disorders according to the DSM-5. The first 19 families were included whose treatment has been completed when the study has started and who had given their informed consent to participate. All of these families had complex problems in different domains that were interrelated and mutually influencing each other.

### Procedure

Group interviews were semi-structured, lasted about 75 min, and chaired by a moderator, the primary researcher or a research assistant, and an assistant. The number of professionals in each group interview was dependent on how many professionals were involved in the treatment of the particular family, varying from two to five. Prior to the group interview, the professionals were informed about the aim and the topics of the group interview. In preparation for the group interview, the electronic case file for the parent and child were studied by the researchers and displayed in a compact chronological overview in a timeline. The moderator presented this at the start of the group interview as a warmup for the professionals. Most of the time, this was the start of a spontaneous discussion between group members.

Interview questions were open, not based on the literature but guided by topics related to an integrated family approach. These were: (theoretical) considerations about the shift from an individual to a family approach in treatment, the efficacy of the whole treatment, which factors in the treatment were helpful and whether any challenges were experienced, the contribution of the regularly multi-disciplinary consultation, the influence of the other colleagues, and critical thoughts about and satisfaction with the treatment.

After finishing each group interview, the moderator and the assistant shared their impressions in a debriefing about atmosphere, content, observations, and differences with previous group interviews. Group interviews and debriefings were audio taped and transcribed and the text was proofread.

The method of constant comparison between the different group interviews was used during the process of data sampling and a few interview questions were added to explore specific topics in more detail. For example, when some professionals were spontaneously talking about what they learned from working with this approach and what it means to them personally, a question about learning was added because it seems an interesting topic. Another adjustment in the interview guideline was made because to understand which processes contributed in the cases in which an integrated family approach did not work out well.

### Data Analysis

The transcripts of the group interviews were anonymized, which means that each professional was provided with a code indicating their discipline, so the analysis process was not influenced by remembering the person. Data were analyzed using grounded theory following the analysis stages including open, axial, and selective coding (31). Atlas-ti 8 software was utilized for coding. During the open coding phase, codes were attached to quotes that could be meaningful in light of the research questions. Subsequently, this process was iterated by comparing new information with old data. In the next phase of axial coding, themes and categories emerged through linking various codes together. These categories provided the building blocks for developing grounded theory. The coding of the transcripts was done by at least two independent researchers (14 by the primary researcher and research assistants and five by two research assistants), who afterwards compared and discussed their coding to achieve consensus. Reliability of coding was assessed by estimating the degree of consensus upon completion of the coding of each transcript. In case of disagreement in the interpretation, when coders could not reach consensus about a part of the text, the primary researchers decided how to code the text. The process of coding includes memoing and writing a debriefing after finishing coding for each group interview. The memos contained information about shared emergent ideas about, perceptions of, and relationships identified within the data, and were used in searching for patterns to achieve a grounded theory.

After 16 group interviews, no new information emerged, but given the wide diversity of the cases three more group interviews were added to control for possible bias. Data sufficiency (34) was achieved when no significant new information emerged during the final group interviews and no new codes emerged during the analysis. The text of the interviews and all codes were read, reread, and compared. From the data, six superordinate categories and nine subordinate categories formed the basis of the emergent theory about key elements and processes which contributed to or impeded the success of a multi-disciplinary integrated family approach conducted by adult and child mental health services.

## Results

The treatment of 19 families was evaluated in group interviews with all professionals who were involved in a substantial part of the treatment of that particular family, except in one group interview. In that one case, a professional who was intended to participate in the interview but was unable to attend at the last minute. We decided to interview her by phone. Of the 17 group interviews, 14 were done in-person, but due to the Covid-19 lockdown, three were online. No difference was found in the quality from the data of the live and online meetings. In two of the 19 evaluations it was impossible to get all the involved professional together. In these two cases, all these professionals have filled in a questionnaire with the same content as the interview topics. Table 1 provides some information about features of the parents and children in the 19 families and how many professionals attended each group interview. Some professionals participated in several interviews because they were involved with various cases. From this table it can be seen that the members of the families who were evaluated are heterogenous regarding their DSM-5 classifications, ages, and duration of treatment they received. Comorbidity is present in 68% of the parents and 37% of the children. We tried to follow the daily practice of complex cases treated by mental health services very closely. In clinical practice there is wide variety in the phenomenology of mental disorders (35) and the contexts of the patients and families. Therefore, this study did not focus on a specific classification to avoid the false impression of a homogeneous group that could have been treated in a uniform way.

**Table 1:** Features of parent and child which treatment was evaluated by professionals in 19 group interviews.

***N***	**Parent**			**Child**			**Treatment period^2^**	**Professionals^3^ in group interview**	
	**DSM-5**	**C**	**Age^1^**	**DSM-5**	**C**	**Age^2^**		**AMHS**	**CAMHS**
01	Personality disorder	1	23	Parent-child relational problem	0	24	10	1	1
02	Personality disorder	4	32	Post-traumatic stress disorder	2	1	24	1	1
03	Personality disorder	1	43	Parent-child relational problem	0	9	18	1	1
04	Personality disorder	1	30	Post-traumatic stress disorder	1	24	27	2	1
05	Personality disorder	1	36	Unspecified neurodevelopmental disorder	1	52	15	1	1
06	Personality disorder	2	18	Unspecified neurodevelopmental disorder	1	24	16	2	2
07	Bipolar disorder	1	33	Parent-child relational problem	0	3	13	3	2
08	Depressive disorder	0	30	Parent-child relational problem	0	60	9	1	2
09	Depressive disorder	1	26	Parent-child relational problem	0	7	20	1	1
10	Depressive disorder	0	39	Parent-child relational problem	0	12	14	1	2
11	Anxiety disorder	0	32	Parent-child relational problem	0	10	8	2	2
12	Anxiety disorder	2	28	Parent-child relational problem	0	4	10	2	1
13	Autism spectrum disorder	2	33	Autism spectrum disorder	1	24	16	1	2
14	Autism spectrum disorder	0	44	Parent-child relational problem	0	11	33	1	2
15	Autism spectrum disorder	1	33	Parent-child relational problem	0	48	15	1	3
16	Post-traumatic stress disorder	0	37	Unspecified neurodevelopmental disorder	1	60	30	1	2
17	Post-traumatic stress disorder	0	38	Parent-child relational problem	1	8	15	3	1
18	Post-traumatic stress disorder	2	27	Parent-child relational problem	0	8	20	2	2
19	Other specified trauma and stressor-related disorder	1	30	Parent-child relational problem	0	12	8	2	1

*C, comorbidity disorders*;

1 *amount in years*;

2 *amount in months*;

3 *categories of professionals: psychiatrist, psychotherapist, psychologist (clinical psychologist, general psychologist), group therapist, family or couples' therapist, nurse (nurse specialist, community psychiatric nurse), professional in home treatment*.

**Figure 1:**
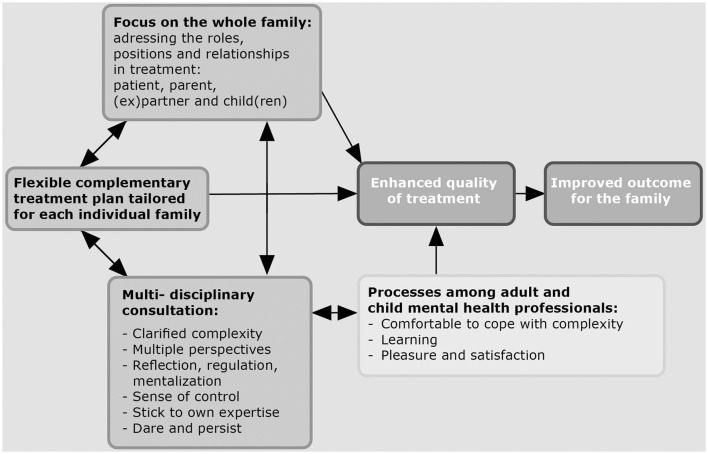
Grounded theory about key elements, processes and benefits of an integrated family approach according to professionals.

The analysis of the data showed that according to professionals, an integrated family approach in mental health care generates value for families, although there were also challenging issues that could pose a threat to treatment success. We found three different important key elements and three processes amongst professionals which led to enhanced quality of treatment and improved outcomes for the family (see Figure 1). The three key elements are: first, the *family is seen as a whole and the distinct roles, positions and interrelationships of the family members are addressed in treatment* by the different services; second, the *treatment plan is flexible, complementary, and tailored*; and third, there is *multi-disciplinary consultation on a regular basis*. The three processes amongst professionals which led to enhanced quality of treatment because of their consultations were: *being comfortable in coping with complexities of problems, learning, pleasure* and *satisfaction*.

The main challenging issues in collaboration that pose a threat to the intended benefits are differences between the AHMS and CAMHS professionals in therapeutic concepts, organization policy, loyalties, need to exchange information, and differences in ideas about realistic targets.

### Key Elements Within an Integrated Family Approach That Contribute to Benefit of the Family

#### Focus on the Whole Family

Professionals considered it to be of value that in the different treatments by the two services (CAHMS and AHMS), the focus was on the whole family, *addressing distinct roles, positions, and relationships*. It especially benefited parents who were able to distinguish between their roles in treatment: The role of an individual with a mental disorder, a parent of a child, and partner or ex-partner. In an integrated family approach, all distinct roles of the adult were addressed. In the treatment of the parent-child relationship at CAMHS, the vulnerability of parenthood and the fear of losing their child through outplacement impacts parents' behavior. While in individual treatment of the mental disorder parenthood is not the focus, this makes the parent more able to work on their own issues in the therapeutic relationship without being anxious about consequences for their parenthood. A psychotherapist (CAMHS) who worked with the parent-child relationship described an example of this as follows:

*I have always felt that she was more socially desirable with me. With you [nurse AMHS] she could also express anger. With me she suppressed that very much from, I suppose, the fear of “what if I show it here?” The consequences for her, whether she could raise her child. And so, it was a good thing that it could all be there*.

#### Flexible Complementary Treatment Plan Tailored for Each Individual Family

The direct benefit that professionals experienced was that they were better able to “*grasp the whole picture*” of the family and the environment because the focus is on the entire system.

In a family of two children aged 3 and 6 years, all members were struggling with autism or ADHD. There were housing and financial problems, and they experienced a lot of stress due to their lack of overview and planning skills. The involved professionals attuned their treatments to each other and prioritized what was most necessary so that the family was not overburdened. The nurse of the mother said:

*Tuning in [with each other] also makes sure that you get an overall picture. I think that's really important. Because if something is going to happen in the treatment of the child, it has an impact on the treatment of the mother. And the way I work with mother affects the treatment of the child. And certainly, if patients don't tell you, if you don't ask about it, it's just possible that you [colleague CAMHS] would have started a whole treatment and I would never have known about it through the mother*.

By attuning the treatment components to each other and tailoring them to the capabilities of the family, an integrated family approach enables a better-suited and improved outcome of treatment. Flexibility and timing were mentioned as essential elements. Sometimes temporizing is needed to give the parent time to become open to learning and taking advantage of an intervention. If this succeeds, therapeutic processes become complementary and reinforce each other, resulting in improved outcome. In one of the families, it took a year before the parents were motivated and there was enough space to start couple-relations therapy. The couples' therapist said:

*It was a good timing when I came in, and I also noticed it was very helpful that we were doing it together as well as each of us doing their own part. Because I wouldn't have been able to do anything with this couple if she [the mother] didn't get healthier and it didn't calm down at home with the daughter*.

The psychologist at AMHS agreed: “*I think we have been achieved this with the flexibility of all the different treatments*.” The psychotherapist at CAHMS added that although their intervention had started with a focus on parent-child interaction, this was not appropriate, and was redirected to a home treatment with a more practical approach aimed at parenting issues and cooperation as parents.

With another family with a baby and a mother with a personality disorder embroiled in a complicated divorce, both psychologists from AMHS and CAMHS adapted their goals and tempo of treatment to the situation. The psychologist at AMHS answered the question of whether she was affected by the treatment conducted by her colleagues as follows:

*I have become less ambitious in my attitude toward the mother. If this is the reality in which she lives and if this is what is going on than a number of things cannot be done now. The mother was also ambitious in terms of education and work. And because I had this contextual information I thought: “Hey, wait a minute, if all this is going on then she has to be more careful on certain points.”*

The psychologist at CAHMS working with another family said: “*We knew from each other what we were doing. I really thought that was an added value…. I knew when the trauma treatment of the mother was going to start and that she needed space for it*.”

Professionals at CAMHS especially emphasized the importance of considering the information from their colleagues about the impact of the mental disorder and capacities of the parent(s) and adjusting their treatment accordingly. A psychotherapist said:

*It was, because in our regular consultation you [nurse AMHS)] told me about her history, that I recognized that I was involved with someone who had a serious chronic mental illness. That did help me to be more careful…. It helped me to determine what I could do and could not*.

#### Multi-Disciplinary Consultation

Multi-disciplinary consultations, in which the information provided by all professionals was brought together, and *clarified the complexity of the family*. All of them had problems in different domains, including the parental mental disorder, the young child's individual problems, and the parent-child relationship. In most of them there were also problems in other facets of the family (the other parent, the partner relation, the other children) or the environment (e.g., financial, housing). With all this shared information and *multiple perspectives*, professionals were able to develop a joint vision, recognize patterns in processes in treatment, and were more able to prioritize and stay on the same track.

In a family with complicated relations and loyalties, the psychotherapist from AHMS became aware through consultation of how she was taken in by idealizing the partner of her patient and that the family dynamic was more complicated than she could see from her position as a therapist who focused on the individual mental health problems.

*I think that if you do it together, you will get a better understanding of such a complicated [family] system*.

It turned out that professionals were easily overwhelmed by the complexity of the families which exceeded their own knowledge and expertise. In one case the mother and her baby were referred, but at the start of the treatment it turned out that the father also was suffering from a mental disorder, and they experienced problems in their partner relationship. Three mental health services (two AMHS and CAHMS), the child protection system, and social services were involved. The grandparents participated in the care of the baby. In the group interview five professionals participated. The couple therapist said:

*There was so much going on. A lot was needed. You could never have done this on your own…. You'd be pulled into a hundred pieces…. All those different contexts and different people seeing different people, saying different things*.

An important process that emerged was the *regulation* of emotions professionals experienced within themselves through the knowledge that another colleague was treating the problems in a different but related domain. Knowing that all risks and problems in the family were addressed by colleagues gives professionals *a sense of control* and supports professionals *in keeping focus on their own expertise and targets*.

A single mother with borderline personality disorder (BPS) and multiple traumas with hardly any support system was referred for treatment after delivery. The involved nurse motivated her patient to contact a colleague from CAMHS because she was concerned about the impact of the mental disorder on the development of the baby and the parent-child relationship. She felt that working with the baby was beyond her expertise and the therapeutic alliance too fragile to allow her to do that:

*She [the mother] had quite a big emotion regulation disorder. And with the baby there, that was a concern for me. So, it was good to know that there was a child therapist involved, who knows much more than I about mothers and infants. … That gave me more peace of mind … When I saw her [the mother] I always thought about that baby. But I don't have to say anything about my concerns about the baby. I could not do that, I know my colleague does. … If I would have said something about it, I would have lost her [drop out]*.

The same experience was brought up by professionals at CAMHS. A mother and her baby were referred for parent-child therapy, after hospitalization of the mother because of severe depression and suicidality. She was involved in a complicated divorce. The involved psychologist said:

*I found it quite complex, their intention to divorce, and it was important for me that you [family therapist] took that piece. It gave me space to really work on that parent-child relationship. But this is also the case with the individual therapy of the mother. I knew she had really been very depressed and suicidal, and it was important to know that this was monitored by my colleague*.

The multi-disciplinary consultations provide professionals *space to think* about the meaning of the problems, to *understand* how they are interrelated, and to think about what the best port of entry for treatment will be to elicit change. A mother with a major depression and her baby had been referred for treatment with an integrated family approach after a crisis period. The professionals felt much pressure from the parents to achieve rapid improvement, which was not a realistic expectation given the severity and complexity of the problems. In the multi-disciplinary consultation this was discussed and the reflection about the meaning of it led to a better understanding. The psychotherapist from CAMHS mentioned this as follows:

*Our regular consultation contributed to a better understanding of the mother's process … her desire to solve things quickly. Her trouble with tolerating that it doesn't work like that. Something did happen there [in the consultation] that we could understand her better and so it gave us a chance to talk about it with her*.

The multi-disciplinary consultation supported professionals in *maintaining their reflective stance* in challenging processes as splitting between the therapists, and handling a fragile or complicated therapeutic relationship by sharing their feelings. In one case, the treatment of the child was on hold because the mother was angry and did not trust the psychotherapist at CAMHS anymore. She was splitting the therapists into good and bad, but this did not lead to disruption between the collaborating colleagues because they discussed this in their consultation. The psychotherapist at AMHS made this a topic in the therapy with her patient. This was important in enabling the psychotherapist at CAMHS to stay involved until she could continue the treatment.

In cases in which professionals experienced powerlessness, friction between them emerged easily, which provoked the risk that these feelings would be transformed into anger at the other colleagues, as stated by the parent-child therapist: “*I think the risk in this case is that you say, ‘Oh, the other person [colleague] is not doing it right. That's the one who has to fix it.’ We all didn't get it solved*.”

Another important result mentioned by the professionals was that they were *daring* to be more directive and confronting in their own treatment because of the involvement of their colleague of the other service. A nurse from AMHS said that she dared to discuss difficult things that she would otherwise suppress because of the vulnerability of the mother, knowing that the stability of the young child is guaranteed by her colleague. “*Well, especially that collaborating, … yes, I tackled more things, dared more. … And I think that otherwise I would have thought ‘that's not possible, because there's a baby’. … I think I wouldn't have discussed certain things*.” The psychotherapist at CAMHS in turn dared to confront more because she saw that her colleague was doing so and it did not lead to disruption in the relationship.

The multi-disciplinary consultation performs a regulatory function when it comes to being *persistent* despite slow progress. The problems, resulting from accumulating and interrelated risk factors in different domains resulting in negative cascade effects, are not easy to change and generally take a long period of treatment, sometimes several years. A mother with a personality disorder lacked feelings of bonding with her 9-month-old baby. She was convinced that her baby did not need her and so absorbed in her own concerns that she was constantly searching for confirmation of her motherhood in the baby's behavior. Consequently, she was not emotionally available enough to the baby, who in turn was avoidant toward her mother which was again a confirmation for the mother that she was superfluous. At AMHS, individual psychotherapy with the mother was conducted in which her own attachment history was an important topic and EMDR was part of her treatment. At CAMHS, videos confirmed to the mother that her baby was avoidant of her (not due to autism, but an attachment issue). She could discuss her feelings about the baby with the therapist and with help she could be emotionally available to her child and sensitive to the child's needs. There were also a few sessions with the parents and both therapists. It took one and a half year to change the beliefs of the mother and to change the patterns in the parent-child interaction. The psychotherapist who addressed the parent-child relationship gave her reflection on this collaboration with her colleague: “*And that we [colleagues] had contact and that we could share encouraged me to keep going, not to give up. Because it was sometimes very difficult, there was so little progress, especially in the first year*.”

### Processes Among Adult and Child Mental Health Professionals That Contribute to Benefit of the Family

The key elements identified above generate processes in the professionals which enable them to enhance their functioning.

#### Comfortable to Cope With Complexity

As illustrated in the above section, by joining the multi-disciplinary consultations, processes among professionals occur in which they felt regulated and calm, making them comfortable in coping with the complexity of the problems in these families.

#### Learning

A specific process mentioned by professionals was learning from colleagues with another area of expertise, as described by a nurse (AMHS): “*I received something extra, because I've also seen your work, and I really liked that. …I've learned a lot more about attachment and about [parent-infant] regulation*.”

In one of the group interviews, the AMHS professional brought up that she had felt uncomfortable giving feedback to her colleague at CAMHS about her treatment which did not match with this mother. The parent-child therapist's reaction was that this was indeed “*hard to take*”, but it had helped her.

#### Pleasure and Satisfaction

In a majority of the group interviews, it was mentioned that the collaboration provides more pleasure and job satisfaction, wherein the following aspects were mentioned: “*It becomes more lively*,” “*The children are in the picture*,” “*It provides multiple perspectives*,” “*More involvement*,” “*More sense of connection*,” “*It is motivating*”, “*That we all did it together, good allocation of tasks*,” and “*More things succeed that otherwise would not succeed*.” Especially professionals in AMHS got a broader view: “*I enjoyed viewing the case together and from different perspectives, discussing the child's interests, possibilities of treatment for the parent(s), the ‘stuck in between’ position of parent-child therapist, and searching for a helpful solution*.”

### Enhanced Quality of Treatment and Improved Outcome for the Families

Overall, professionals indicated that by using an integrated family approach, involving the three key elements, together with their strengthened functioning, both the quality of treatment and the outcome for the family is improved. They perceived treatment as having improved impact with their treatments compared to working separately. The following results were reported: reduction of symptoms of the parental mental disorder, and improvement of quality of the parent-child relationship, partner relationship and family relationships.

The professionals discussed this point and concluded that treatments with an integrated family approach carried out by adult and child mental health services needs time and effort involving multi-disciplinary consultation, but in the end will be more efficient and produce an improved outcome for the family. A nurse treating the mother in a complex situation with problems in all domains made this point as follows:

*What I learned from it is that I continue focus on collaboration and communicating about when you run into things. Even though there is not always time or opportunity to do so. I don't think we would have gotten that far without this collaboration*.

A therapist of a mother expressed her view of the efficiency of collaborating with professionals with different areas of expertise as follows: “*When I started treatment that way with you [colleagues at CAMHS], I had a lot more background information as well. I didn't have to find out the same things you did*.”

Not surprisingly, many professionals mentioned the *preventive impact* of working simultaneously with the adult and the parent-child relationship, because this is an important goal of this integrated family approach. With prevention, mostly the impact on the development of the child is considered: “… a*nd where we hope that there will be a solid foundation [for the infant], I think we can be satisfied that we have made a nice contribution with a lot of work, and with a lot of waiting and acceptance of pace”*.

In some group interviews, it appeared that in general there is more attention from those at AMHS for the children of the patient because of the existing collaboration. In addition, it was mentioned that more families could benefit from this integrated family approach.

One participant, a psychiatrist (AMHS), mentioned that an eye opener for her was recognizing the importance of looking beyond the individual to the whole context. They jointly chose a different port of entry in treatment than the guidelines prescribe. Instead of treating the chronic traumas of the mother, they chose parenting and the parent-child relationship as the port of entry in line with the request for help, and she was surprised that the duration of the treatment was shorter and the outcome of the treatment was better than she had expected at the start:

*And that is so miraculous to me, that if you go beyond the individual and just go to the [family and social] context, treatment can have a completely different focus, as a result of which something happens to the individual which has completely surprised me. […]*

*Well, I also think that from an individual viewpoint and just follow the guideline then “We have to do this and this and this,” while if you take a contextual viewpoint then there are so many more factors you can act on, which can also lead to a lot of change in the individual. That is what I have learned from this case*.

### Challenges in Collaboration by Adult and Child Mental Health Services

Professionals experienced various challenges in carrying out their treatments with an integrated family approach involving both adult and child mental health services. The challenges mentioned by professionals are related to the traditional differences in therapeutic concepts and organizational policy between the two services and loyalty of professionals to their own service and patient.

In a few cases the professionals struggled during treatment with the differences in the therapeutic concepts whereby on the one hand from an individual point of view the adult patient is seen as an autonomous individual with their own responsibility and on the other hand from a family approach perspective the adult is seen as a young parent with a dependent infant. For instance, professionals at AMHS emphasized patients' own responsibility for not showing up for a therapy session and the importance of their accepting the consequences of that: if that is repeated, the therapy will be terminated. Professionals at CAMHS were more likely to reach out in the interest of the young child.

There was a particular group interview regarding a case of a 18 years old mother who, on the one hand, was eager for trauma treatment, but on the other hand, did not attend her appointments regularly. The professionals discussed this, and they conceptualized the problem differently from each other. The therapists from AMHS defined it as a motivational problem: “*She had not really committed to her treatment*”. The professionals from CAMHS defined her behavior as a product of her being a mother who developmentally was still a teenager, with planning problems which required more support from professionals to get the therapy started, for example through home visits. In their multi-disciplinary consultation, they struggled to decide regarding the port of entry. The professionals from CAHMS preferred to give priority to treating the traumas of the mother based on the idea that she was so preoccupied with her traumatic experiences that it impeded her from connecting with her child. The professionals from AMHS, in contrast, experienced that it was beyond their control to get the mother to show up for her sessions. Because there was a problem with parenting and the parent-child relationship, this is arguably a better port of entry. They all experienced a lot of powerlessness being caught in the dilemma between ending treatment because there did not seem to be a good context for treatment and on the other hand feeling motivated to help dependent young children.

In another case, the professional from AMHS mentioned that if she had involved the mentor of the mother-child home in an earlier stage, she would have had information about the abuse that was going on, but it was against the therapeutic concept to do so in an early stage of treatment:

*She [mentor] said, “There had been abuse for some time, why didn't you respond?” And I thought: but I didn't know that at all. … In hindsight, I thought: maybe I should have had this mentor in the room much earlier and looked more closely at “How is it really going?” or “What is your impression as a third party?” With us, a third party [outside the organization] comes in very hard. In general, I agree with that … we think the patient's own control is important… but with this mother I think afterwards “She [the mentor] had so much information.”*

The loyalty to one's “own service” in this case was particularly focused on by the professionals from AMHS. On the one hand, they wanted to be loyal to the rules of the service they worked for, and on the other hand, they felt the pressure of the interests of the children that the colleagues from CAIMHS pointed out.

Different views regarding what were realistic targets in treatment are also mentioned in a case where the treatment at AMHS and CAMHS started at various times. The professional who was just entering treatment was more optimistic than the one who had already had a long-term therapeutic involvement with the family, which caused some strain in their joint effort.

Another point particularly brought up by professionals at CAMHS was they felt a greater need to exchange information compared to their colleagues at AMHS because they needed to know what they could expect and ask from the parent. This dynamic was confirmed by the colleague at AMHS who felt no need to get frequent information about the progress of the parent-child therapy. It was sufficient to know that the child was seen in treatment. Moreover, the professionals from AMHS felt a great responsibility to protect the privacy of their patient, which sometimes prevented them from sharing information easily, despite parental consent to do so.

The collaborative relationship of professionals in treatments including parent and child can fall under pressure due to loyalties, especially if there are young children. The dependency and vulnerability of the young child easily leads to identification with the child, resulting in blaming the parent, who is the patient of the therapist at AMHS. If this dynamic is not addressed, it will isolate the therapist in a bond with the patient and withdrawal from joint treatment may occur. In one case this did occur. The therapist of the mother felt her patient was judged and she had to defend her: “*It seems to me that the mother's problems were blamed on her, that she shouldn't do certain things as a mother. While I think yes, she is also a patient…. The children had to be protected, but that atmosphere was too much for me*.”

All these mentioned challenges interfere with fruitful collaboration, and clarify what conditions are needed to prevent this from happening. Professionals struggling with these challenges highlighted the importance of commitment of all involved professionals to the family approach, taking more time for reflection, and ensuring that the interests of all family members are represented in the multi-disciplinary consultation.

## Discussion

The analysis of the data showed that according to the professionals, an integrated family approach in mental health care generates value for parents with mental disorders and their young children. This study revealed three important key elements that led to enhanced quality of treatment and improved outcomes for the families. The first key element is that the family is seen as a whole, and the distinct roles, positions and interrelationships of the family members are addressed in treatment by the different services. The second key element is the flexible, complementary, and tailored treatment plan, and the third key element is the multi-disciplinary consultation on regular basis.

The focus on *the family as a whole* provides information about the parental mental disorder, functioning in the parental role and family relationships, and the functioning of the child. This complementary information helped professionals to grasp the whole picture. Sharing information and observations in the *multi-disciplinary consultations* made it easier to recognize and understand patterns and themes. This contributed to making a better assessment of which goals were viable and how they could tailor their treatment to each individual family. *Flexibility in attuning their complementary treatments* to each other and *tailoring them to the capabilities of the family members* enabled the whole treatment to be applied better to the needs of the whole family. Because of these key elements in an integrated family approach, the professionals perceived their quality of treatment as enhanced, resulting in improved outcomes. The mentioned key elements prevent fragmentation of treatment, which is mentioned as an important barrier in providing integrated care (36, 37), and overburdening of the family.

As a result of these key elements, *processes* emerged in which professionals felt more comfortable in coping with the complexity of the problems in the family. They experienced a sense of control and regulation in conducting their treatment while facing a lot of problems in different, related domains in family life and the environment. They experienced support from their colleagues, knowing that they were working on related domains. This study showed that the multi-disciplinary consultation plays an important part in this. The process of working together resulted in professionals being able to keep their focus on their own expertise and targets, to maintain their reflective stance, and to think about and understand challenging processes in the therapeutic relationship and the dynamics between the therapists involved. The capacity to understand behavior of self and others in terms of internal states such as feelings, desires, and needs is called mentalization (38). Maintaining the mentalizing stance of collaborating professionals within a team is mentioned by Nijssens et al. (39) as an important capacity in challenging interactions in treatment, through which “coherence and consistency of the treatment” remains ensured (p. 85). Furthermore, professionals felt encouraged to be more directive and confrontational in their treatments and holding on and moving forward despite the treatment path being “long and bumpy.” In addition, professionals expect prevention of adverse outcomes, especially for the young child, which makes them consider their work to be more meaningful. In most evaluations, professionals told us they felt more pleasure and satisfaction in working together in the treatment of the whole family and learning from each other. All these benefits for the professionals indicate an indirect *benefit for the family* that received their treatment of better regulated and more focused professionals. The professionals' feelings of self-efficacy is one of the identified facilitators in providing integrated care (36).

Another benefit for the family mentioned by professionals is in addressing the distinct roles of patient, parent, and (ex)partner in different treatments. The impact of the mental disorder is not limited to the individual functioning of the adult, but also affects the relationship with the children and the partner and, conversely, the context of the family may affect the course of symptoms. The involvement of professionals from both AMHS and CAMHS in the treatment honored the distinct roles and relationships in family life and allowed the parent to feel more comfortable about paying attention to their functioning as an individual and in relationships within the family.

Our findings about the importance and benefit of addressing the distinct roles and positions in the family are in line with previous research of family focused practice. Parents often have concerns about the effect of their mental disorder on their parenting and relationship with, and development of their children (40), but do not easily bring this up in their own treatment for fear of losing custody of their child (41). In addition, mental health professionals at AMHS are not trained to treat parenting and child development issues (42). These barriers experienced by parents and professionals can be overcome by adopting an integrated family approach.

The novel element in this study is the integration of multi-disciplinary treatments conducted by professionals from two services, AMHS and CAMHS, targeting the whole family. However, although this offers many opportunities to increase the quality and impact of total treatment, it is also more complicated, particularly because this entailed a team around the family in which professionals had not previously worked together and did not share the same therapeutic views (37, 43).

Besides the benefits, we also found *challenges* in conducting treatment of the whole family which pose a threat to the collaboration and a joint focus on the treatment of involved professionals of AMHS and CAHMS. These challenges are differences in therapeutic concepts, organization policy, loyalties, need to exchange information, and ideas about realistic targets. Sharing information between professionals from both services in a multi-disciplinary consultation is one of the key elements of treatment embracing an integrated family approach. If a professional is reluctant to do so due to personal values, their colleagues are limited in their ability to tailor the treatment to the capabilities and circumstances of the family. In our sample, we saw a tendency for CAMHS professionals being more in need of information regarding making decisions about how to direct their treatment than their colleagues at AMHS. This is a direct consequence of the different goal statements of the two services. Treatment of the young child is impossible without the parent (44), whereas treatment for the adult could be done without involving the children.

Another challenging factor was the different therapeutic concepts and policies between the services. This is related to the traditional difference in focus between CAMHS and AMHS, and the responsibility felt by the professional. On the one hand, the individual focus of AMHS views the patient as autonomous and with responsibility for their own lives. On the other hand, the focus of CAMHS is on the child and the parent-child relationship, and the professional feels a certain responsibility for the development of the child. This is not in a legal sense but arises from a sense of commitment to the vulnerability and interests of the young child. These differences pose a challenge and can potentially give rise to conflicting views on issues regarding port of entry and termination of the treatment. The loyalty of the professional to their own patient and own service is easily triggered by controversy and may keep them away from being committed to the whole family.

The above-mentioned processes which threaten collaboration between AMHS and CAMHS are in line with previous research (45, 46).

## Limitations

Some limitations of our study concern the sample of families whose treatment was evaluated by the professionals. First, the recruited professionals were from two mental health services in a small part of the Netherlands. This is because there are no other mental health organizations with a comparable liaison between AMHS and CAMHS embracing an integrated family approach wherein professionals are facilitated to do so. Second, we included all professionals involved in the treatment of families that occurred during our study, and we finished data collection after data sufficiency, which possibly results in bias in terms of the specific characteristics of the sample. Although there was sufficient diversity among the included families, we cannot exclude bias partly because the study took place in a geographically small area. Third, professionals and researchers are part of the same organization, and in certain cases, they know each other, which carries the risk of their not being willing to speak out openly. Fourth, in this study we have only used one method and one source, the experiences of professionals, which is a limited foundation for a grounded theory approach presenting a concept of a theory about an integrated family approach in mental health care. This paper is part of a broader study, with the goal to build a model grounded in data of different sources as the literature, experiences of patients, observations, and measurements of treatment outcomes. In the additional studies different methods will be used to establish a more elaborate theory.

### Implications for Clinical Practice

This exploratory evaluation suggests that treatments using an integrated family approach carried out by adult and child mental health services working together is of value for the families involved by empowering professionals from the different services to collaborate with one another. The experiences of professionals in this study were helpful for informing the management and the workforce of mental health services about which key elements and processes are associated with an integrated treatment approach will benefit parents and children and their relationships. By paying attention to the challenges and threats that often emerge in these treatment programs, they are better able to create conditions in which professionals will be family focused in their treatment.

An interesting finding is the great benefit of the *multidisciplinary consultation* of involved professionals, on the one hand the conducting of complementary well-tailored care for each individual family and on the other hand for professionals to be able to cope with complexity in the family. These findings may seem contradictory considering the current call for patient involvement in shared decision making (SDM). SDM is a concept wherein decisions are made based on interaction with the patient, regarding which treatment has the best evidence and is most appropriate with respect to patients' values and preferences (47, 48). However, multi-disciplinary consultation and SDM do not exclude each other. The multi-disciplinary consultation enabled the professionals to see all aspects of the whole family. Because of this, the professionals are better able to assist patients and parents to make shared decisions.

## Conclusions

The experiences of professionals in this study indicate that using an integrated family approach in treatment could be of benefit for the families involved, although there are challenging issues that could pose a threat to it. The key elements that provide benefits for the family are the focus on the whole family, flexible complementary tailored treatment, and multidisciplinary consultation. Professionals indicate that because of these key elements, they were more comfortable coping with the complexity of problems in the families they treated, and they perceived that this led to better quality of treatment and resulted in improved outcomes for the family. The key elements providing benefits and the challenging issues can be understood as a recommendation to the managers to enable professionals of adult and child mental health services to collaborate and discuss their issues, all for the benefit of families.

## Data Availability Statement

The raw data supporting the conclusions of this article will be made available by the authors, without undue reservation, and on request by email to the first author.

## Ethics Statement

The studies involving human participants were reviewed and approved by Medical Ethics Review Board at the University Medical Centre of Utrecht in Netherlands. Written informed consent to participate in this study was provided by the participants and participants' legal guardian/next of kin.

## Author Contributions

HS put the article into writing and collected the data and conducted the data analysis with MS. All authors contributed to the article in having read and commented on the manuscript text and approved the final version of the article.

## Conflict of Interest

The authors declare that the research was conducted in the absence of any commercial or financial relationships that could be construed as a potential conflict of interest.

## Publisher's Note

All claims expressed in this article are solely those of the authors and do not necessarily represent those of their affiliated organizations, or those of the publisher, the editors and the reviewers. Any product that may be evaluated in this article, or claim that may be made by its manufacturer, is not guaranteed or endorsed by the publisher.

## Supplementary Material

Supplementary Figure 1 Grounded theory flow chart (49).
