# Supplementary figures and images for: Integrated Family Approach in Mental Health Care by Professionals From Adult and Child Mental Health Services: A Qualitative Study
Source: Front Psychiatry. 2022 Apr 28;13:781556. doi: 10.3389/fpsyt.2022.781556 (PMC9096092; doi:10.3389/fpsyt.2022.781556)

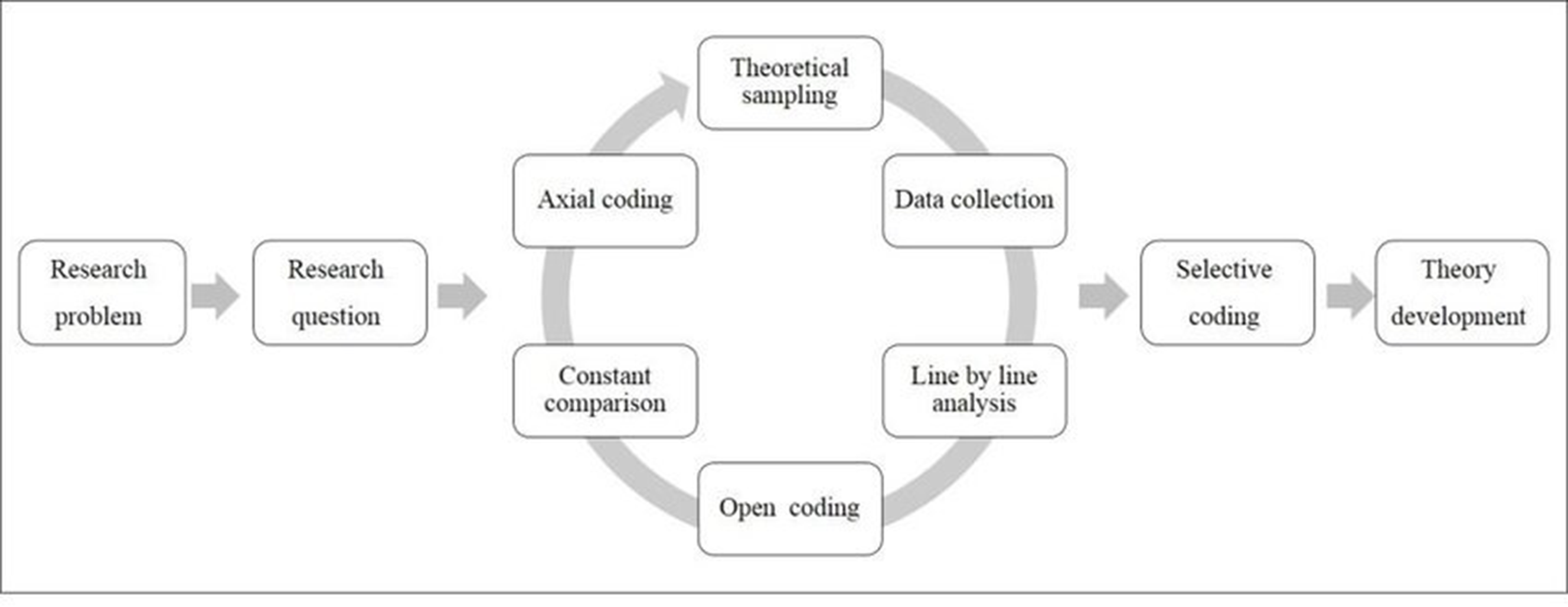

Supplement: Supplementary Figure 1 — Grounded theory flow chart (49). [file Image_1.PNG]
